# Directed coupling in multi-brain networks underlies generalized synchrony during social exchange

**DOI:** 10.1016/j.neuroimage.2022.119038

**Published:** 2022-05-15

**Authors:** Edda Bilek, Peter Zeidman, Peter Kirsch, Heike Tost, Andreas Meyer-Lindenberg, Karl Friston

**Affiliations:** a Wellcome Centre for Human Neuroimaging, Institute of Neurology, University College London, 12 Queen Square, London WC1N 3AR, United Kingdom; b Department of Psychiatry and Psychotherapy, Central Institute of Mental Health, Medical Faculty Mannheim, Heidelberg University, Square J5, Mannheim 68159 , Germany; c Department of Clinical Psychology, Central Institute of Mental Health, Medical Faculty Mannheim, Heidelberg University, Square J5, Mannheim 68159, Germany

**Keywords:** Hyperscanning, Dynamic causal modeling, h-DCM, Joint attention, Social interaction

## Abstract

Advances in social neuroscience have made neural signatures of social exchange measurable simultaneously across people. This has identified brain regions differentially active during social interaction between human dyads, but the underlying systems-level mechanisms are incompletely understood. This paper introduces dynamic causal modeling and Bayesian model comparison to assess the causal and directed connectivity between two brains in the context of hyperscanning (h-DCM). In this setting, correlated neuronal responses become the data features that have to be explained by models with and without between-brain (effective) connections. Connections between brains can be understood in the context of generalized synchrony, which explains how dynamical systems become synchronized when they are coupled to each another. Under generalized synchrony, each brain state can be predicted by the other brain or a mixture of both. Our results show that effective connectivity between brains is not a feature within dyads *per se* but emerges selectively during social exchange. We demonstrate a causal impact of the sender's brain activity on the receiver of information, which explains previous reports of two-brain synchrony. We discuss the implications of this work; in particular, how characterizing generalized synchrony enables the discovery of between-brain connections in any social contact, and the advantage of h-DCM in studying brain function on the subject level, dyadic level, and group level within a directed model of (between) brain function.

## 1 Introduction

### 1.1 Hyperscanning

Recent work in social neuroscience has identified neural signatures that relate to human coordination and social exchange (Jirsa and Kelso, 2005; Kelso, 1995; Nordham et al., 2018). In neuroimaging, these insights rest on simultaneous functional brain recordings of multiple subjects while they interact socially, representing a move towards real life contact, termed *hyperscanning*.

At a neural level, correlations between brain regions from individuals engaged during social inference can be observed, which are significantly stronger during immediate social contact (Bilek et al., 2015). In prior work, we demonstrated that these correlations can be found in the right temporo-parietal junction (rTPJ), a core region of the social brain network, implicated in the central coordination and control of social information and behavior (Krall et al., 2014; Schurz et al., 2014). During cooperative interaction, rTPJ networks - engaged in both subjects’ brains - show a degree of coherence in their activity (Bilek et al., 2015; Goelman et al., 2019). For other forms of social interaction, other brain regions such as the medial prefrontal cortex (mPFC) may contribute to neural synchrony (for review, see Redcay and Schilbach, 2019). A growing body of evidence speaks to the relevance of neural coupling (i.e., synchronous brain activity between brains, independent of the modality or computed measure) between individuals in diverse forms of social contact, with half of the existing peer-reviewed literature being published within the past three years (Pubmed search for *hyperscanning*, last query 04.05.20). For example, neural synchrony underlies verbal communication, is associated with successful information transfer (i.e., understanding) between individuals (Silbert et al., 2014; Stephens et al., 2010) and predicts the learning success in interpersonal teaching settings (Pan et al., 2018; Stolk et al., 2014; Zheng et al., 2018). Moreover, the bonding level between subjects is reflected in peak synchronization (Kinreich et al., 2017; Pan et al., 2017), and mother-infant empathy and learning are associated with neural synchrony during their interactions (Levy et al., 2017; Reindl et al., 2018). This kind of synchronization is thought to be a key aspect of therapeutic alliances (e.g., Koole and Tschacher, 2016; Kupper et al., 2016), and interestingly, within-group structure as well as between-group conflict relate to synchronization between members (Jiang et al., 2015; Yang et al., 2020). A few studies have demonstrated clinical value of brain synchronization in the form of a neural synchronization deficit in social interaction disorders, i.e. Borderline Personality Disorder (Bilek et al., 2017) and Autism (Tanabe et al., 2012). In line with this, interaction-based phenotyping of psychiatric patients might be particularly useful for understanding the (social) brain and its disorders, as well as in the development of personalized treatments (Leong and Schilbach, 2019; Schilbach, 2016).

In summary, these studies suggest that neural synchronization between brains is a fundamental function of the human brain that is related to key characteristics of social interaction and its success. This makes neural coupling a promising new target for both the mechanistic understanding of interpersonal exchange, and the development of novel interventions to support social functioning.

### 1.2 Correlation and causality

Inter-brain associations are often measured in terms of statistical dependencies among two or more fMRI timeseries. Common examples in the time domain include (cross-) covariance and (cross-) correlation; their counterparts in the frequency domain are the cross-spectral density and coherence, respectively (see Fig. 1 of Friston et al., 2014b). Moving beyond instantaneous associations among timeseries, (non-parametric) Granger causality describes temporal precedence among pairs of timeseries, an approach that has been successfully applied in the study of inter-brain associations (Schippers et al., 2010). These multi-variate *data features* or *descriptive statistics* are static transforms of the data, and the analysis of which is typically referred to as *functional connectivity* in the neuroimaging literature (or *directed functional connectivity* in the case of Granger causality). However, such measures do not explain how putative generalized synchrony between brains is caused. Correlations are a descriptive feature of the data, not the mechanism generating the data. On the contrary, in the study of social interaction and related disorders, we are concerned with the causal effect one brain may have on another. It is important to understand whether brain states are shared between interacting individuals, the direction of how these effects are shared (e.g., which direction of effects is relevant at a particular point in time, or during a specific joint action; thus inferring causality within a network), when they are shared (e.g., when we cooperate vs. when we do something separately but at the same time/place), and how they are affected by individual and interaction-related factors (e.g., similarity between partners, individual personality traits, interaction styles). This aids a mechanistic understanding of social interaction on the brain level, and the development of interventions to facilitate neural coupling; for example, between patients and clinicians in psychotherapy, where optimal neural synchrony may relate to mutual understanding and therapeutic alliance building.

Furthermore, correlations between simultaneously acquired data are interpreted as information flow or the emergence of shared brain states (Redcay and Schilbach, 2019). However, this interpretation is challenging: all correlative measures of synchronization between brains can be partly induced by a shared sensorium. In other words, when subjects experience similar sensory inputs, the evoked and induced responses will be correlated to a greater or lesser degree. This is the same problem that faces characterizations of *functional* connectivity between regions within the same brain. Researchers have tried to remove the correlations between sensory bound brain responses that confound tests for (and interpretations of) synchronization of a nontrivial kind, for example through permutation approaches or task control conditions (Bilek et al., 2015; Koike et al., 2019) (for a review on fMRI hyperscanning analysis methods see Czeszumski et al., 2020). However, this procedure does not inform whether a connection between brain systems may be assumed. We have to test empirically whether neural coupling *between* brains provides a ‘better’ explanation for the neuroimaging data than single-brain networks (i.e., whether between-brain models yield a more accurate prediction of the data and we find higher evidence for such models than for single-brain models). In this context, the best explanation or model is the one that maximizes the probability of observing the data given the model – i.e., the model evidence. This can always be decomposed into the accuracy of the model minus its complexity. For example, we can expect the activity of individuals watching the same movie to correlate, because the identical data is processed in two brains. Single brain networks will be most efficient in explaining the correlated neuroimaging time-series, because they are the least complex networks that provide the most accurate prediction of data. For social interaction, we find correlations between time-series that may not be fully accounted for through shared perceptual input (Redcay and Schilbach, 2019). We wish to quantify this specific effect, and we wish to infer the effective connectivity of our network.

In this paper, we address these issues using measures of *effective* connectivity and Bayesian model comparison in the setting of dynamic causal modeling (DCM). DCM can be used to distinguish potential causes of correlations, while and quantifying the evidence for competing hypotheses about the network architectures generating these correlations. Both, shared sensory input and effective connectivity between brains could result in correlated time-series. To disambiguate between synchronization and shared input as potential causes of correlation, the models considered here include both so that they directly compete in explaining variance. Specifically, a large number of different parameter combinations are examined and compared in their accuracy of predicting the data. This allows one to compare the evidence for models with and without effective coupling between brains, in the context of the shared input. We thus use model evidence to determine which network structure is optimal in predicting the data. Correlated neuronal responses become the data features that have to be explained by models with and without between-brain (effective) connections. However, our main interest lies in the examination of the winning model. After model estimation, DCM provides parameters that quantify the causal and directed effect one brain region exerts over another. This kind of modeling thus offers a causal explanation of how the observed data were generated, and how the brain systems exchange states during social contact.

From previous hyperscanning work we hypothezise that a model that includes connections between the brains of interacting individuals will perform better in explaining the data than models that do not include between-brain connections.

### 1.3 Generalized synchrony

Conceptually, the approach introduced in this paper is grounded on the notion of *generalized synchrony* or synchronization of chaos (Breakspear, 2002; Jafri et al., 2016; Schiff et al., 1996; Schumacher et al., 2012). Generalized synchrony refers to the characteristic behavior of loosely coupled dynamical systems, where one system trains the dynamics of the other and *vice versa* (Hunt et al., 1997). Perhaps the earliest (and most intuitive) example of generalized synchrony is the observation by Huygens that pendulum clocks suspended from the same beam will ultimately come to oscillate in synchrony (Huygens, 1673). This is a universal phenomenon that is well established experimentally and well-understood theoretically, in the context of coupled dynamical systems. Formally, it is best understood in terms of loosely coupled chaotic dissipative dynamical systems, where the coupling causes the states of both systems to synchronize on what is described mathematically as a *synchronization manifold* (Breakspear, 2002). Technically, this synchronization manifold lies in the joint (state) space of both systems and attracts the dynamics of both to a low dimensional attracting set (the synchronization manifold),. When the systems that are coupled have sufficiently similar dynamics (i.e., the functional form of their equations of motion are similar), identical synchronization emerges, and the dynamics of one system can be predicted by the state of another. This is the only long-term solution to the joint equations of motion. Indeed, this is how generalized synchronization is usually inferred; i.e., by asking whether knowing the state of one system enables us to predict the dynamics of another (e.g., as overviewed in Jiruska et al., 2013). Under identical synchronization, there is a simple mapping between the states of one system and the other, which renders this predictability symmetric. The form of this dynamic coupling is exactly the same used in DCM; namely, the flow of neuronal states in one brain region is affected by the states of another (Friston et al., 2014c). The move we make in this work is to consider regions from two brains (using hyperscanning data, h-DCM). Crucially, generalized synchrony rests on the notion of loosely coupled oscillators (e.g., two clocks suspended from the same beam). When applied in the context of neuronal dynamics, the oscillators become highly nonlinear and hierarchically structured neuronal oscillators (Buzsaki and Draguhn, 2004; Fries, 2005; Lisman and Buzsaki, 2008) that constitute the neuronal dynamics of two brains that are coupled by and during social interaction.

Crucially, generalized synchronization can only occur if there is some formal or structural similarity between the coupled systems. Our basic premise here is that the brains of two participants can only become meaningfully coupled via generalized synchrony when they share the same sort of dynamical structure. Indeed, this is exactly the assumption made in formal simulations of communication using active inference (Friston and Frith, 2015a). In other words, if two brains are trying to infer and predict each other, then this inference is facilitated when they become synchronized, i.e., show a generalized synchrony. In the context of active inference, this generalized synchronization underwrites nearly all forms of communication that rest upon a shared narrative and inference process (Friston and Frith, 2015b; Frith and Wentzer, 2013). In this work, we try to reproduce this generalized synchronization in an experimental (hyperscanning) paradigm and evaluate the degree of synchrony in terms of effective connectivity between two brains using h-DCM.

The mathematical notion of generalized synchrony clarifies the biophysical implementation of between-brain coupling. The very existence of a synchronization manifold implies that we can replace the states of one brain with the states of another brain, when predicting the (subsequent neuronal) dynamics. This means that generalized synchronization is an emergent property that makes it look ‘as if’ there is some latent effective connectivity between the two systems in question. Consequently, a simple explanation for the dynamics of two systems that show generalized synchronization can be articulated in terms of effective connectivity.

There is a subtle aspect to this use of effective connectivity, which rests upon the modeling of (neuronal) time series. In brief, inference about connectivity - both within and between brains - means finding the architecture with the greatest evidence. Evidence is the difference between accuracy and complexity; where complexity is the degree to which estimates of coupling diverge from prior expectations, before seeing any data (Penny, 2012). When two brain systems are (identically) synchronized, we obtain the same prediction of brain dynamics using the states of a single brain or a mixture of both. Hence, our single and dyadic brain models attain similar accuracy. Model selection can therefore be based on finding the least complex architecture. Notably, the least complex case is when both brain systems contribute equally to the prediction (i.e., two parameters attain half the value of a model with a single parameter). This means that the simplest explanation for generalized synchrony (under suitable prior beliefs about coupling), is an effective connectivity between brains. Interestingly, this is exactly the same mathematical truism that licenses the assertion that ‘there are no true models’ (Litvak et al., 2019).

In summary, this paper illustrates a characterization of - and evidence-based test for - generalized synchrony in hyperscanning using h-DCM and Bayesian model comparison. It is motivated by the fact that the most likely explanation for generalized synchrony is the presence of latent effective connections between two coupled dynamical systems—that exist due to a synchronization manifold. This means that we can test for the presence of generalized synchrony by comparing models with and without effective connections between two brains.

In what follows, we demonstrate the application of these procedures to a hyperscanning experiment using functional magnetic resonance imaging (h-fMRI), in which we deliberately introduced asymmetry in dyadic interactions, in terms of switching the roles of a sender versus receiver. The task incorporates asymmetry, as it provides information to one participant (sender), but not the other (receiver), and then forces the exchange to complete a trial. We may therefore hypothesize that at least one of the between-brain connections should reach from the sender to the receiver. By switching task roles we can test this hypothesis in both directions from the individuals point of view. This kind of asymmetry, in the context of generalized synchronization, is known as a skew-product system. Our hypothesis was that in one role or another, we could provide substantial (Bayesian) model evidence for a dynamical architecture that involved directed between-brain connectivity. As such, this provides a procedure to determine if observed functional connectivity between brains is, or is not, mediated by the kind of synchronization, attunement or alignment that underwrites a shared narrative or processing of sensory exchanges. Notably, such directed connectivity would be specific to epochs where one individual exerts influence on the other (e.g., by sharing information). It would not be present at every moment or in any form of interactive task.

Following previous work on two-person data, we aimed to replicate between-brain connections between interacting individuals. In addition, we hypothesized directed connectivity from the sender to the receiver of information, which cannot be differentiated by correlative approaches.

Our focus in this paper is on procedures and techniques. We therefore restrict ourselves to an analysis of normal subjects, paying special attention to the analytic details. In subsequent papers, the approach will be adopted to make inferences about differences in between brain coupling associated with psychopathology and inter-subject variability.

## 2 Materials and methods

### 2.1 Sample

We examined a sample of 120 healthy subjects aged 27.5 ± 5.2 years with a mean education of 12.4 ± 0.9 years (76 females, 44 males). Subjects participated in the study as 60 randomly assigned same-sex pairs (mean within-pair age difference 5.4 ± 4.5 years, education difference 1.1 ± 1.5 years). Prior to participation, subjects were screened to ensure they had no history of neurological or mental illness, pregnancy, and conformed to MRI exclusion criteria. The study protocol was approved by the Ethics Committee of the University of Heidelberg, for which subjects provided written informed consent. Data from 18 dyads had been part of a previously published sample (Bilek et al., 2017).

### 2.2 Hyperscanning protocol

Data from dyads were acquired simultaneously at two 3T Siemens Trio MRI scanners. As described in detail in Bilek et al. (2015), scanners were directly connected by optical fibers to ensure data transmission in real time (temporal delay < 1.5 μs). Prior to the MRI measurement, subjects were instructed separately in the task and completed sample trials until the task was fully understood. In addition, they were trained in performing eye movements while keeping the head still. Dyads met briefly in person before scans, but were not given the chance to speak to each other (the task introduction was repeated by staff, and subjects were chewing on a cotton ball for oxytocin sampling). Scanners were assigned randomly to subjects, and a live video stream of the partner's face was provided throughout the measurement.

### 2.3 Task

**Fig. 1:**
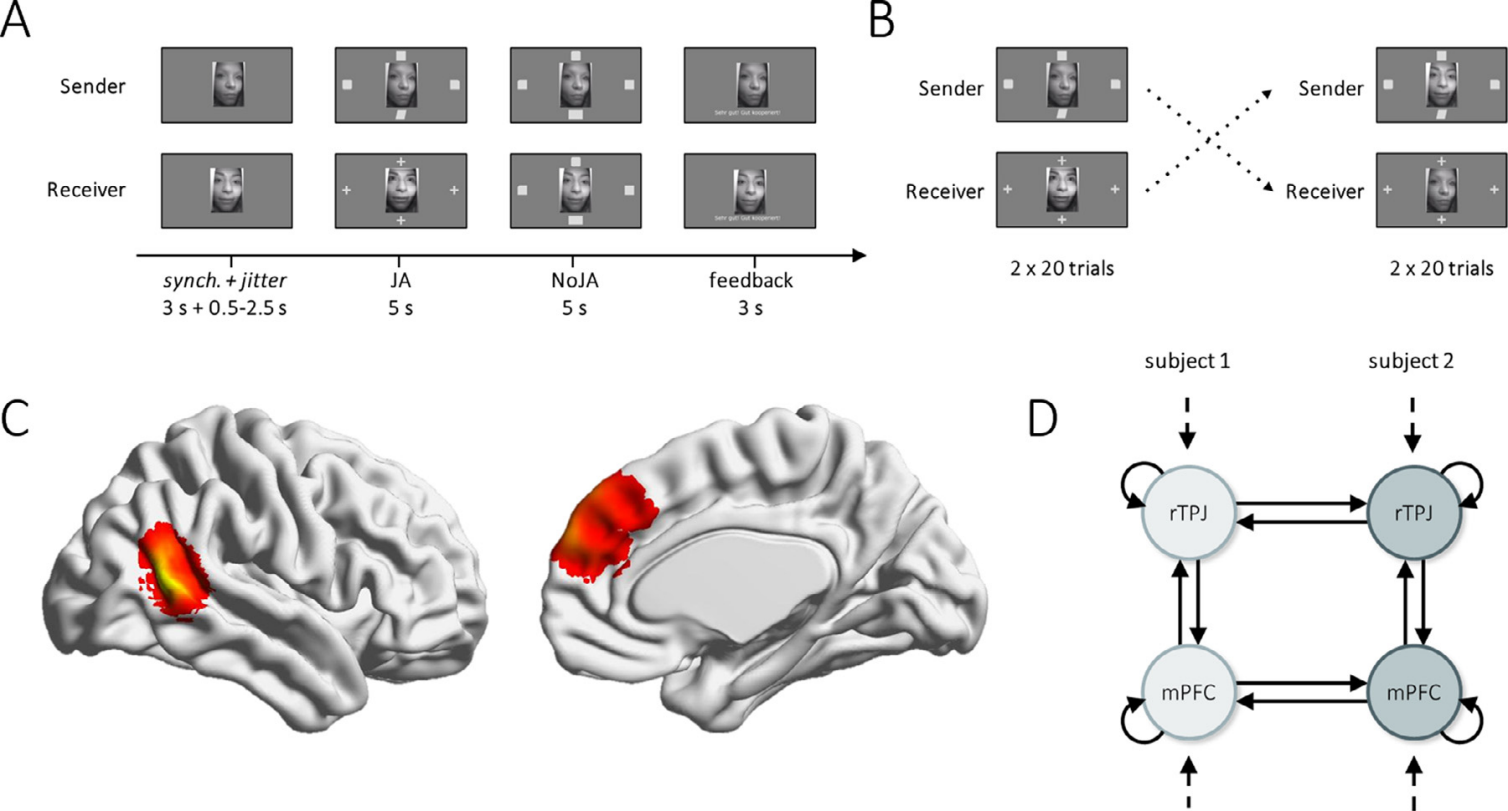
Task structure and specification of the two-brain dynamic causal model (h-DCM). A. Illustration of task trials. **B.** Illustration of the task design. Subjects switched task roles (sender/receiver) after 20 trials. **C.** Summarized individual seed regions from *N* = 120 subjects (60 dyads), rTPJ (left), and mPFC (right), respectively. **D.** h-DCM architecture for two subjects. Time series from both subjects of the dyad were entered into one DCM. Between-brain connections were allowed between the same regions from each subject (horizontal lines). All within-brain connections were included in the model (vertical and curved solid lines). Driving input was received by all four regions (dashed lines), and all connections were modulated by joint attention (solid lines).

In a joint attention task, subjects were required to press a button corresponding to a target stimulus location indicated on the stimulus presentation screen (i.e., left, right, bottom, or top; Fig. 1A) by a particular shape (e.g., square). This information was revealed to one subject only (the *sender* of information), who had to indicate the correct response to the partner (the *receiver*) via eye saccades towards the target location. Receivers were instructed to identify the target location by following the partner's eye gaze. Trials were rated as successful if both subjects pressed the target button on a 4-button diamond shaped response device. Hence, for successful trial completion, subjects had to engage in joint attention. To examine non-JA task-effects, we included a trial phase of individual performance (NoJA), where both subjects received the information about the target location, and therefore completed the trial individually without calling upon communication through eye gaze. Phases of interaction (JA) between the subjects and phases of individual performance (NoJA) alternated for a total of 40 trials.

Target locations were pseudo-randomized between trials, while the remaining three stimulus locations were filled with distractor shapes (e.g., parallelogram, rectangle, square with rounded or cut corners). Receivers viewed four distractor shapes (plus sign) during JA trials. Live video transmission was provided continuously over the entire task, centered on screen, and task stimuli were arranged at the respective positions around the video image. The full task included 40 trials in two scan blocks (alternating phases 40 × JA and 40 × NoJA; 5 s each), followed by a performance feedback (3 s). Task roles (sender/receiver) switched after the first half of trials, so that each subject performed both task roles across the measurement (Fig. 1B). The total task time summed to 651/656 s (triggered/triggering scanner).

Seed regions were visualized with the BrainNet Viewer (Xia et al., 2013). JA, interaction phase; NoJA, individual performance phase; rTPJ, right temporo-parietal junction. mPFC, medial prefrontal cortex. h-DCM, dynamic causal modeling for hyperscanning.

### 2.4 fMRI data acquisition and pre-processing

All data analysis was conducted using Statistical Parametric Mapping software (SPM12, www.fil.ion.ucl.ac.uk/spm/software/spm12/). Scripts for the extension of standard DCM functions to h-DCM models are available from the corresponding author. FMRI data were acquired with the following parameters: TR = 1550 ms, TE = 30 ms, FOV = 192 mm^2^, 28 slices, 4 mm thickness, 1 mm gap, flip angle 73°, 423/420 volumes (triggering/triggered scanner). For preprocessing, images were realigned to mean image, slice time corrected, normalized to standard stereotactic space (as defined by the Montreal Neurological Institute), and smoothed using a Gaussian kernel filter with 8-mm FWHM.

### 2.5 Subject level: analysis of brain activity and seed region selection

We examined brain responses to task conditions in a classical general linear convolution model (GLM) analysis for the purpose of selecting regions of interest and extracting time series for subsequent connectivity analysis. Brain areas that were included in the dynamic causal modeling were examined for task-related activity.

For each subject, a random effects model was specified, comprising five task-related regressors. To differentiate sender and receiver-specific responses, we included one regressor modeling all JA phases for the sending role (JA-send), and a separate regressor for the receiving role (JA-receive). In addition, one regressor included all NoJA. Two regressors modeled cue onset and button responses as events (duration = 0 s). In addition, six movement regressors (of no interest) were entered into the model, corresponding to three rotation and three movement planes. To remove slow frequency drifts, data were high-pass filtered with a cut-off of 128 s. For each subject, contrast images comparing interaction with individual performance [JA-send+JA-receive > NoJA] were calculated.

Based on previous work (Bilek et al., 2015, 2017), we hypothesized effective connectivity between rTPJ of dyads to emerge during social interaction, and selected this region as our first Volume of interest (VOI). A functional segregation of rTPJ has been proposed, comprising a posterior part involved in social cognition and mentalizing, and an anterior part involved in attention (Bzdok et al., 2013b; Mars et al., 2012). Our studies have found posterior rTPJ to host between-brain effects, and defined the region through an empirical mask provided by Bzdok et al. (2013b). In addition, we included mPFC due to its involvement in many social processes, such as mental state inference, social decision making, and importantly, triadic attention (Bzdok et al., 2013a; for a recent review, see Redcay and Schilbach, 2019; Saxe and Baron-Cohen, 2006). MPFC was defined based on definitions within the automatic anatomic labelling atlas (Tzourio-Mazoyer et al., 2002). Summarized masks of the seed regions are shown in Fig. 1C. Notably, we selected these VOIs following our *a priori* hypotheses and interest in between-brain function. However, based on task-related brain activation and other research questions, other brain regions could be considered for analysis in future studies.

To test whether VOIs exhibited relevant activation during interaction, we examined brain responses at the group level with a one-sample t-test, using the contrast [JA-send+JA-receive > NoJA] and a whole-brain FWE-corrected threshold of 0.05. However, we find task-related activity is not imperative in the identification of between-brain connectivity, as we are testing for coupling between brains that might not rest upon stimulus bound responses. Furthermore, prior work has shown that connectivity can be observed in brain regions that do not show significant task related effects (Gerchen and Kirsch, 2017). Future work may therefore select VOIs based on *a priori* hypotheses and meta-analyzes rather than task related responses.

For h-DCM, subject time series were extracted with the VOI tool implemented in SPM, from voxels that conforms to the following criteria: (1) exceeded a liberal statistical threshold of *p* < 0.01 uncorrected (to exclude uninformative voxels) and (2) were within a 6 mm sphere centered on the subject-specific peak (within 16mm of the group peak). Time series were summarized within each region by their first principal component and corrected for uninteresting covariates such as motion.

### 2.6 Dyad level: dynamic causal modeling

H-DCM entails important advances for the inference of neural coupling: we examine effects on the level of neural populations instead of BOLD responses (which serve as a proxy to neural activity), we infer effective connectivity instead of correlation between ensuing time series, and we examine the direction of connectivity between those brain regions.

The task incorporates asymmetry, as it provides information to one participant (sender), but not the other (receiver), and then forces the exchange to complete a trial. We therefore hypothesized directed connectivity from the sender to the receiver of information, which required a separate modulation of the first and second task block to account for the role switch within the task (subject 1 may, for example, perform as sender in the first task block and as receiver in the second; subject 2 of the same dyad will take the complementary role in each block).

For each dyad, a (deterministic) DCM was specified based on the recommendations in Zeidman et al. (2019a), comprising nodes of rTPJ and mPFC of both subjects (4 seed time series; Fig. 1D). In this model, the rate of change of neural activity of each brain region at a specific point in time ($\dot{z}$) can be expressed as a function of the experimental input u and the effective connectivity between and within the brain regions $\theta^{n}$. This is approximated by a neuronal state equation - or equation of motion:

(1) $$\dot{z}=\left(A+\sum_{j=1}^{m}u_{j}B^{\left(j\right)}\right)z+Cu$$

Parameters in matrix A (size n×n) characterize the average connectivity over the experimental conditions. As shown in Fig. 1D, we hypothesized bi-directional connections between regions within the same brain and inhibitory self-connections for each region, which controlled their sensitivity to inputs (i.e., neural gain or excitatory/inhibitory balance). In addition, we allowed homologous brain regions to be connected across the dyad. This was not a prerequisite for the method, but was motivated by theoretical, empirical, and methodological accounts that speak to the connection of the same region in two brains for our data. First, we followed the notion of generalized synchrony that two systems become coupled via generalized synchrony when they share the same sort of dynamical structure. The dynamical structure will differ more between different brain regions in different brains, while we may assume closer relations between the same brain regions. Similar notions have been brought forward by Bolis and Schilbach (Bolis and Schilbach, 2020) in the form of a match or sufficient similarity between interaction partners as the basis of neural coupling. Secondly, studies on cell recordings in rodents revealed specific subpopulations to encode self and other's behavior, but those subpopulations were part of the same brain area (Kingsbury et al., 2019; please see Section 4.5 for more details on animal studies). In addition, prior work on neural coupling during JA and gaze cueing tasks usually reported correlation between the same brain regions (e.g., aside from our own work, Koike et al., 2015; Saito et al., 2010) (reviewed in Redcay and Schilbach, 2019). Lastly, the introduction of additional parameters increases the model complexity by parameter_count*condition_count*dyad_count. It is therefore recommended to formulate parsimonious models, as long the network is a plausible representation of prior knowledge. This was the case for our study, but can be tested formally using Bayesian model comparison as described in Section 2.8 below.

A connection can be removed or switched off by setting the prior variance to zero during model specification. Otherwise, to include a connection in the model, the prior variance is defined as non-zero (1/64 Hz).

The matrix u comprises all experimental or exogenous input to the network. Input can be specified as driving input to the brain regions and eliciting neural responses (e.g. the main effect of task), or as modulator, representing context or condition specific changes in effective connectivity. We defined three vectors as driving input based on the subject-level GLM regressors. First, we specified one input, representing all trials from all conditions (main effect of task; JA-send, JA-receive, NoJA). In addition, we included an input capturing all button responses from the subjects, and one capturing cue onsets. We chose to mean-center the inputs, so that estimated parameters in A represent the average connectivity across conditions and parameters in B represent changes from that average.

Elements in $B^{\left(j\right)}$ (size n×n) represent context sensitive changes in effective connectivity due to modulation by condition j. In the DCM, we specified the three task conditions as modulators, JA-send (for subject 1), JA-receive (for subject 1), and NoJA, respectively. By defining the sender in the first block as subject 1, we were also able to examine changes of effective connectivity over time. We allowed all connections to be modulated by all experimental conditions. Finally, the matrix C (size n×J, for J experimental inputs) parameterizes the effect of driving input on each region. We allowed driving input in all four regions.

As described in detail by Zeidman et al. (2019a), connectivity parameters in DCM are expressed in units of hertz, as these units represent rates of change. Between-region connections describe how much faster or slower the rate of decay in a brain region is as a result of an incoming connection. Self-connections control how long it takes for neural activity to return to baseline, e.g., due to endogenous self-inhibition.

After specification, dyad-level models were inverted (i.e., estimated), to identify the posterior density over parameters that achieved the best fit to the data (accuracy), while penalizing complexity. This is scored by the model evidence, which is used to compare competing models. An approximation to the log model evidence is the free energy F, which can be written as:

(2) F≅lnp(y|m)=accuracy(y|m)−complexity(m)

Where y is the data and m is the model, defined in terms of which parameters are included in the model (i.e., free parameters with non-zero prior variance). On the dyad level, the complexity is the Kullback-Leibler-divergence between the estimated parameters and their priors. For the following group level (Section 2.7), the complexity combines the dyad level complexities and those of the group model, i.e., the difference between group-level GLM parameter priors and the estimated parameters (Friston et al., 2016). The process of choosing the model that provides the greatest evidence or highest free energy is termed Bayesian model selection.

### 2.7 Group level: parametric empirical bayes

In order to estimate connectivity effects at the group-level, we used a hierarchical regression model over h-DCM connectivity parameters, namely, Parametric Empirical Bayes (PEB; Friston et al., 2016; Zeidman et al., 2019b). At the first level of the hierarchical model, the observed fMRI data $Y_{i}$ for subject i are modeled under a dynamic causal model (hey denoted by Γ) with connectivity parameters $\theta_{i}^{\left(1\right)}$ and observation noise $\varepsilon_{i}^{\left(1\right)}$ (Eq. (3), bottom row). Parameters that are not modeled at the group level (hemodynamics and noise) are treated as fixed effects. At the group level, all dyad-model connectivity parameters are collated into a parameter vector $\theta^{\left(1\right)}$ and subsequently modeled with a GLM, with group-level parameters $\theta^{\left(2\right)}$, design matrix X and random effects $\varepsilon^{\left(2\right)}$ (Eq. (3), top row).

(3) $$\theta^{\left(1\right)}=X\theta^{\left(2\right)}+\varepsilon^{\left(2\right)}$$

$$Y_{i}=\Gamma \left(\theta_{i}^{\left(1\right)}\right)+\varepsilon_{i}^{\left(1\right)}$$

Our hypotheses concern the condition-specific effects of social interaction. Hence, our main interest lies in the first level (within-dyad) parameters of the B matrix, encoding condition-specific changes in connectivity (Eq. (1)). However, these parameters mediate condition-specific changes, relative to the average connectivity (A matrix). We therefore estimated two PEB models: one for the average connectivity across all task conditions, and the other testing for condition specific changes in connectivity.

### 2.8 Model specification, comparison, and reduction

The group-level design matrix X was defined by first specifying the second level (between-dyad) design matrix $X_{B}$, which had one row per dyad and one column per covariate. The first column was a vector of ones (i.e., the sample mean or conserved effects across dyads). Subsequent columns represented covariates or group membership (i.e., differences between dyads). This was duplicated over connectivity parameters by taking the (Kronecker) product with the identity matrix $I_{e}$ (where e denotes the number of effective connectivity parameters):

(4) $$X=X_{B}⊗I_{e}$$

After specification of the design matrix, we inverted (i.e., estimated) the model and estimated the free energy of each second level model. We considered multiple plausible (between-dyad) design matrices with different combinations of common covariates in classical GLM analyzes of fMRI data, and selected the model which best explained the data. This process is termed Bayesian model comparison. We used standard procedures for Bayesian model inversion (fitting) and model comparison implemented in the DCM framework within the SPM12 software. In particular, model inversion was performed using the variational Laplace scheme (Friston et al., 2007), which employs variational Bayesian procedures that eschew the need for sampling. For each subject's model, this returns an approximation of the log model evidence, referred to as the variational free energy or evidence lower bound, which serves as the basis for Bayesian model comparison (Kass and Raftery, 1995). To rapidly compare models at the group level, we used an analytic approach known as Bayesian model reduction (Friston et al., 2018), which is a generalization of the Savage-Dickey density ratio. For more detail on the implementation of the DCM framework and the relevant Matlab functions, please see Zeidman et al., (2019a; 2019b).

Specifically, three models were compared to a model without between-dyad covariates: one model controlling for pair age and within-pair age difference; a second model controlling for age, age difference, and sex; a third model controlling for age, age difference, sex, as well as pair education (sum of years) and within-pair education difference. It is noteworthy that the addition of a covariate generally increases the model's complexity (because the number of estimated model parameters increases), which lowers the model evidence (i.e., accuracy - complexity). However, if the covariate is meaningful in explaining the data, this increases the accuracy. If the increased accuracy outweighs the penalty of higher complexity, we observe an increase in model evidence.

For the selected model, we used a particular form of Bayesian model comparison termed Bayesian model reduction as implememented in the SPM software package (Friston et al., 2018). Effectively, this procedure prunes away redundant parameters that do not contribute to model evidence. Note that we only included plausible connections in the full model; therefore, we treated all reduced model as being equally probable *a priori*. Bayesian model reduction enables an automated search over many possible reduced models for comparison of each models evidence as described above. The procedure iteratively discards parameters but stops whenever the exclusion of a parameter reduces the model evidence. The result is a Bayesian model average over the 256 most probable models, weighted by their evidence.

Finally, we tested whether the resulting between-brain connectivity changed over time. This was in line with observations in prior work (Bilek et al., 2015), which speak to an increase in synchronization over time. This was implemented using a Bayesian contrast, which compares condition-specific parameters (as in a classical GLM contrast), and furnishes a probability density over the difference between condition specific effects on various connectivity parameters. We compared the first and the second block of JA, specifically the connection from sender to receiver.

## 3 Results

### 3.1 Social interaction related brain activity

To examine task-related brain activity on the group level, we performed a one-sample t-test on subject level images contrasting joint attention with individual performance [JA-send+JA-receive > NoJA]. Specifically, we examined rTPJ and furthermore mPFC responses to JA performance (Fig. 1C). Both brain areas showed significantly higher activity during social interaction, compared to task phases where no interaction occurred. This was examined in a one-sample t-test (*T*_rTPJ_ = 10.9, *P* < .001; *T*_mPFC_ = 14.2, *P* < .001; FWE-corrected for the whole brain).

### 3.2 Dynamic causal model selection and average connectivity

For each dyad, a (deterministic) DCM was specified, comprising nodes of rTPJ and mPFC of both subjects (Fig. 1D). On the group-level, we entered the h-DCM connectivity parameters into a hierarchical PEB model and estimated one model for the average connectivity across all task conditions, and a second model testing for condition specific changes in connectivity.

**Fig. 2:**
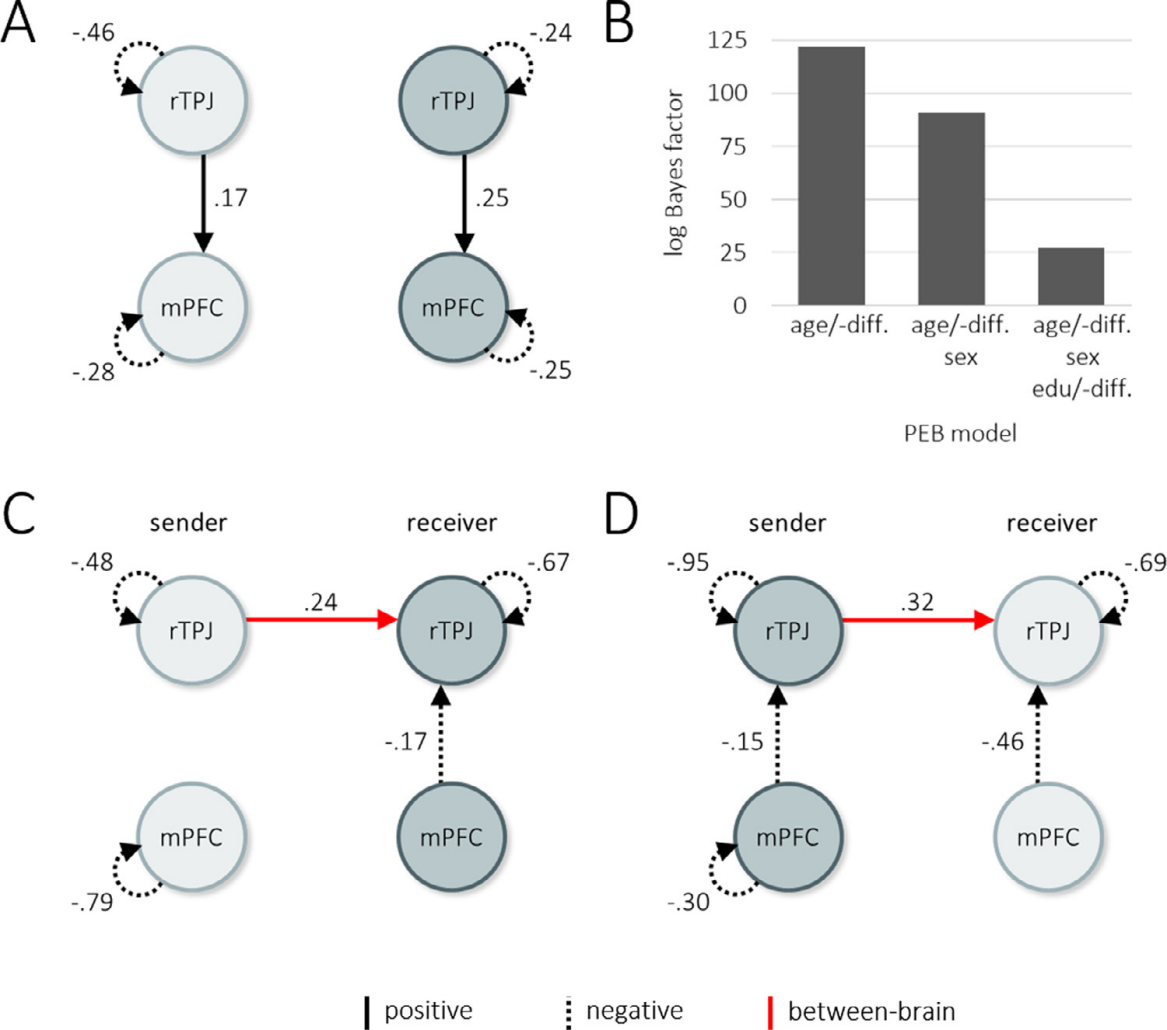
Analysis of effective connectivity during social exchange. A. Average effective connectivity across all task phases (parameter matrix A of the h-DCM neural model). **B**. Bayesian model comparison. PEB models comprising different combinations of between-subject covariates were examined in terms of their contribution to model evidence (i.e., model accuracy minus complexity) compared to the model without covariates. The left model (controlling for pair age and age difference) showed the highest increase in model evidence (i.e., highest log Bayes factor relative to the model without covariates) and was selected for further analysis. **C**. Effective connectivity during cooperation in the first task block (parameter matrix B). **D**. Effective connectivity during cooperation in the second block after task role switch.

Fig. 2A illustrates the group mean of each connection strength across all examined conditions (matrix A). This showed positive effective connectivity within-brain from rTPJ to mPFC during the task (effect size across conditions subject 1 = .17 Hz, subject 2 = .25 Hz) in both task roles. All four seed regions exhibited task sensitivity, i.e., showed a main effect of task in form of an increase of activity (-.46, self-connections are defined as inhibitory, negative parameter values therefore reflect disinhibition). No between-brain connectivity contributed to the model evidence in this model average across conditions and effects.

Panels A, C and-D show Bayesian model averages after Bayesian model reduction of parameters. Circle colors indicate different subjects. Numbers indicate estimated effect size of the respective connection. PEB, parametric empirical Bayes; age/-diff, mean pair age in years and within-pair age difference; edu/-diff, mean pair education in years and within-pair education difference; rTPJ, right temporo-parietal junction; mPFC, medial prefrontal cortex.

### 3.3 Social interaction related connectivity

We used Bayesian model comparison to consider multiple plausible between-dyad design matrices with different combinations of common covariates for fMRI data. All models that included covariates showed greater model evidence compared to the model without covariates (Fig. 2B). We selected the model correcting for pair age and within-pair age difference, which had the highest increase. The inclusion of education and sex, did not provide sufficient additional model accuracy to compensate for the increased model complexity, leading to reduced model evidence (i.e. accuracy – complexity).

After Bayesian model reduction, we found a between-brain effective connectivity that was specific to social contact, in both task blocks, and from the sender's to the receiver's rTPJ (effect size first block = .24, second block = .32; Fig. 2 panels C and D, red solid arrows). This corresponds to our previously reported measure of neural coupling that was based on correlated ICA timeseries (Bilek et al., 2015, 2017). In addition, we found decreased connectivity from mPFC to rTPJ in the receiver in both blocks of JA (effect size first block = −.17, second block = −.46). Self-connections in rTPJ of both subjects and mPFC of the sender showed negative values (effect sizes rTPJ between −.95 and −.48; mPFC = −.79 and −.30), which translates to relative disinhibition during joint attention. No condition specific modulation was observed in mPFC of the receiver, indicating that activation in this region was not selectively changed during joint attention. The covariates pair age and within-pair age difference had no relevant effect on parameters.

The between-brain rTPJ parameter values were descriptively higher in the second task block, compared to the first block . To quantify the probability of this difference, we used a Bayesian contrast comparing parameter estimates from the first and second block. For our connection of interest, the probability of a difference was 73.2%. There was therefore only weak evidence for an increase in effective connectivity over time.

## 4 Discussion

In this study, we examined effective between-brain effective (directed) connectivity during social exchange. We aimed to disambiguate between possible explanations of previously reported correlations between neuronal responses during interpersonal interactions. Our aim was to test for latent causal connections mediating generalized synchrony in a brain dyad.

### 4.1 Between-brain connectivity

Using h-DCM, we specified a formal hypothesis about the directed neural connectivity between the brains of interacting subjects. During model estimation, the effect size of these effective connections (i.e., the causal effect one region has on another), was estimated. The (Bayesian) selection of models was based on scoring evidence for competing models (e.g., models that do or do not include a connection between brains), and in a last step we used Bayesian model reduction to remove all connections that did not contribute to model evidence. Our results disclosed one connectivity parameter in the resulting Bayesian model average; namely, a connection between the brains. This represents effective connectivity between brain systems and explains the generalized synchronization of dynamics observed during social interaction. Crucially, this effective connection was necessary to explain the data, when modeling the shared perceptual experience of the dyad.

The causal connection was present between rTPJ of both subjects. This region has been highlighted by a large number of studies in terms of its involvement in higher social functioning. It is thought to host the ability to infer the social intentions and goals of others, i.e., mentalizing and theory of mind, enabling empathy and the selection of appropriate social behavior (Molenberghs et al., 2016). Our work extends this range of functions to a central role in the synchronization of partners during social exchange. Notably, no other between-brain connectivity parameter contributed to the model evidence, although this might differ depending on the task, the form of interaction, and the model.

Our approach tested connections between brain regions bi-directionally, from subject 1 to subject 2 and *vice versa*. However, following the task design, we expected relevant information to flow from the sender to the receiver, since the sender is initially in possession of the information and is tasked with transferring it to the receiver. Indeed, this was confirmed by the direction of effective connectivity we found, represented by a parameter quantifying the effective connectivity from the sender's rTPJ to the receiver's rTPJ. This proof of principle extends previously reported measures of neural coupling (Bilek et al., 2015, 2017) by the differential effects from sender and receiver brain systems, which is important for the application of h-DCM models in the study of naturalistic social exchange. In the presented JA task, the direction of connectivity is somewhat intuitive, which may not be the case other forms of interaction. However, directionality of effects is particularly important for the understanding of complex, dynamic and non-symmetrical forms of social contact, for which h-DCM models infer *directed* neural processes that underwrite social exchange. Notably, our task included a switch of task roles by participants after the first task block, so that each individual played both roles with the same partner. We applied the same h-DCM model to both task blocks to examine within- and between brain function. Role-specific effects from the two blocks can therefore be viewed as a repetition of results within the same sample.

Clinical relevance of misattunement on the brain level has been highlighted in both conceptual work (Schilbach, 2016) and empirical studies (Bilek et al., 2017; Tanabe et al., 2012). While a match or sufficient similarity between interaction partners across multiple time scales and modalities has been proposed as the basis of neural coupling (Bolis and Schilbach, 2020), mental disorders of social interaction may in fact be characterized by a misalignment of two brains in dyadic interaction (Bolis et al., 2017). It is therefore important that future work on interaction difficulties examines the causal and temporal course of successful interaction in order to disentangle contributions of joint movement, actions, shared goals, task representation, and biological signals such as synchronized heart rate and eye blinks. On the level of neural activity, h-DCM contributes to this by allowing us to quantify between-brain associations over and above shared input, and relate other joint characteristics to the underlying effects between brains, including the potentially affected directionality of connectivity. This is crucial for novel interventions aiming to facilitate social skills, which commonly focus on a single individual (i.e., through work on one individuals’ thoughts, goals, or by practicing social behavior such as small talk). Dyadic interventions may impact the interactive process itself, for example through the achievement of optimal between-brain effective connectivity, to enable mutual understanding, shared goals, and ultimately successful contact.

Alignment can only occur in reciprocal dyadic settings, at least by allowing mutual viewing of the partner's mimic responses (Gallotti et al., 2017). This directly relates to another advancement of social neuroscience, namely the implementation of ecologically valid experiments in hyperscanning settings, which is a departure from previous laboratory approaches (for an extensive discussion, see Schilbach et al., 2013) and provides important novel leads for mental health research (Schilbach, 2016). Especially when combined with neuroimaging modalities that allow for more bodily movement such as fNIRS and EEG, we may examine increasingly complex social events, such as intergroup conflict (Yang et al., 2020), or classroom learning (Bevilacqua et al., 2019). H-DCM examines brain networks on the level of neural activity, not on the level of the data and the modality used. Its application is therefore not limited to fMRI hyperscanning and our neural models may be applied to EEG and fNIRS data as well.

The application of h-DCM is beneficial, as our results rest on directed causality (in a control theoretic sense). By using dynamic causal modeling and Bayesian model comparison, we empirically test for directed coupling and quantify evidence for between-brain connections that represent meaningful synchronization during interactions. Moreover, these methods enable one to directly compare and test different underlying network architectures, their relations to social interaction processes, and – in future work – deviations from normal functioning in individuals who are challenged, for example, by social interaction disorders (e.g., personality disorders, autism, schizophrenia). Hence, we have the means to examine social processes within subjects, within dyads, and between dyads in a framework that has been well established for single subject analyzes. This further speaks to the usefulness of h-DCM in the analysis of between-brain associations using social interaction timeseries.

### 4.2 Directed connectivity in the absence of physical neural connections

The notion of alignment in social interaction predates its study in social neuroscience. Moreover, it has been described for multiple levels of observation, ranging from motor and behavioral alignment to shared intentions and mental states (Gallotti et al., 2017). Through higher forms of alignment (with or without shared goals), a joint action task will be represented differently (Sebanz et al., 2006), because partners align their representations through joint actions and reciprocal exchange. Models of social exchange have proposed a reciprocal circuitry between interaction partners as the scaffold, and alignment may emerge on different levels of observation (Redcay and Schilbach, 2019). However, on the brain level, directed effects between brains cannot rest upon structural connectivity between separate individuals, which may be implicitly assumed for single brain networks. In dynamic and engaged social exchange, reciprocal interactions allow for multi-level alignment of interaction partners (Gallotti et al., 2017; Schilbach et al., 2013), for which we propose the brain synchronization processes involved in cooperation as follows: neural signals in individual A may represent his or her current mental or motivational state, decision making and more, which ultimately result in behavior. Behavior can be viewed here as the output of an otherwise closed (brain) system of A. In the reciprocal model however, this output also serves as the observations or perceptual input for individual B's otherwise closed system. The information is processed and affects individual B's thoughts and decisions, leading to relevant brain activity. Consequently, this also results behavioral output of B, which is observed by A, and so on. In the course of the interaction, we may witness an alignment of higher representations such as shared goals or mutual understanding, quantified on the neural level by specific between-brain connectivity. Prior work supports this assumption by demonstrating the presence of between-brain coupling in a variety of settings such as conversation (e.g., Spiegelhalder et al., 2014; Stolk et al., 2014), cooperative goal achievement (Bilek et al., 2015; Bilek et al., 2017), and learning in school settings (Pan et al., 2018; Zheng et al., 2018). This view aligns with predictive coding models of social exchange and resulting generalized synchrony (Friston and Frith, 2015a), which may be used to extend social neuroscience experiments by means of simulations (Friston and Frith, 2015b) to study the behavior-guiding beliefs that generated observed data or other unobservable phenomena.

Work on invasive brain recording in rodents aid us in understanding the biophysical mechanism that allows for between-brain coupling Kingsbury et al. (2019). recorded prefrontal activity during a competitive encounter of rats, as well as in freely roaming animals. They found that neural coupling depends on processing of social information within subsets of neural populations that are selectively modulated by the partner's behavior (in this study, populations encoding push or retreat behavior, among others). Each individual therefore holds a representation of its own, as well as the partner's behavior, and subsets of neurons encode specific behavior patterns for each agent (e.g., push, approach, retreat, neutral). During interaction, those representations become aligned and neural activity becomes synchronized. Moreover, specific behavior interactions contribute to the coupling (i.e., push behavior cells in dominant animals with retreat cells in subordinate animals), which relates to our modeling of asymmetry in joint attention. Strikingly, the resulting neural coupling also predicts future interactions. The authors conclude that neural coupling is therefore not only an emerging trait during interaction, but relevant for ongoing interactive behavior. Studies in monkeys found similar specific encoding of a partner's movements by neuronal subpopulations, and demonstrated dependency on the distance to an interaction partner during a passive observation task (Tseng et al., 2018). Local field potentials in bats during natural interactions revealed a dependency of between-brain correlation to being in the same social environment, and engagement in social interaction for high correlation (Zhang and Yartsev, 2019).

Notably, these models highlight the importance of disentangling the brain signals due to shared perceptual input from between-brain coupling resulting from social exchange. During social interaction, subjects partly share sensory input. In the study of between-brain function, we are concerned with neural signals that result from the social contact, not the sensory input. A few studies have addressed this issue: in prior work, we permuted pair assignments to quantify baseline neural coherence between individuals that is due to the same, time-locked perceptual processing. This distribution of coherence between non-interacting random dyads thus represents the null hypothesis (i.e., all correlation is due to shared input). Any between-brain correlation shown within interacting dyads, that is significantly higher than baseline can be attributed to the social exchange (termed neural coupling, for details see Bilek et al., 2015). Other studies make use of task control conditions with slight variations of the respective interactive paradigm. For example, Koike et al. (2019) included a 20s shift of the live video from the partner, therefore prohibiting recurrent interaction and introducing a social ‘offline’ context, which resulted in a breakdown of between-brain coherence measures. The presentation of prerecorded videos from a task partner is a variation of this solution. In h-DCM, contributions to common neural activity are modeled by separate modulating parameters, therefore specificity to social contact (i.e., the respective task phase) is implicit to the between-brain parameters. Similarly, we have to explicitly test whether a connection between the individual brains is a better representation of the observed data (i.e., provides a better prediction) than models without this connection. Only if models that include the between-brain connections outperform models without them, a between brain connection should be assumed. The Bayesian model reduction step of our analysis examines all connections and combinations of connection in this manner, and removes parameters whose inclusion do not increase the model evidence as compared to models without them. In our analysis, most between-brain connections were removed by this step (in addition to within-brain connections). We may therefore state that a model that assumes a connection between rTPJs of interacting individuals is the best explanation of our data (given our initial model).

### 4.3 Generalized synchrony

Dynamic causal modeling conceptualizes neural states in a brain region as a result of induced changes in activity and causal effects of other brain regions. In other words, it parameterizes the flow of neuronal states in the state space as a function of neuronal states in the region in question, and elsewhere. In this work, we demonstrated the ensuing dynamics are effectively caused by regions in different brains.

From the perspective of theoretical neurobiology: in active inference, the brain is assumed to constantly predict states of affairs in the world. During social cognition, we use information gathered about the other, their goals, and possible action sequences to select our own responses. We therefore predict each other's behavior and install such predictions in our own choice behavior. During prosocial interactions, this prediction is mutual, i.e., both partners try to predict each other, which forms the basis of a shared dynamical structure through shared inference processes. Generalized synchrony signals this shared inference, since it can only occur when the predictive processes align, i.e., the interacting subjects achieve generalized synchrony, under the same generative model of their shared sensorium. This notion has been formulated in prior work using simulations of social exchange (Friston and Frith, 2015a; Friston and Frith, 2015b). Our experimental results support this formulation of belief updating and underlying brain dynamics. Future work on the application of active inference models and simulations to (behavioral and neural) hyperscanning data may advance our understanding of social exchange along these lines.

The vast majority of publications reporting neural synchrony are based on data from cooperative, hence positive, interactions, e.g., learning, mutual gaze, or motor coordination. In these types of contacts, subjects often engage with each other at the same time (e.g., joint attention as either sender or receiver vs. individual task completion). Other forms of interaction impose a strong reciprocal asymmetry, non-complementary behavior, and phases of social exchange vs. individual performance may not necessarily predefined (e.g., during sequential decision making, feedback, and waiting in a trust game task). However, we have no reason to believe that neural coupling is limited to (experimentally predefined) positive or complementary contacts. Speculatively, this publication bias may have resulted from a methodological aspect. Correlation quantifies identical synchronization, in the sense that the two brain systems evolve identically over time. This symmetry is increasingly violated with progressing complexity of the interaction, separation of action policies, and in non-cooperative exchanges, leading to non-identical behavioral and neuronal dynamics. Consequently, measures of coherence will eventually fail as signatures of synchrony. Generalized synchrony however, denotes the predictability of one dynamic system through another, when both are loosely coupled. Hence, it is a useful construct for understanding any form of real-world social contact.

### 4.4 Within-brain connectivity

The h-DCM included connections that quantify changes of activity and connectivity within each subject's brain system over time. Here, we found a relative disinhibition in brain activity in rTPJ of both subjects, and in the mPFC of the sender. Both regions therefore increased their responses during interaction. No specific modulation was observed in mPFC of the receiver during joint attention, suggesting that this region is not engaged specifically during cooperation. These results correspond to prior findings on task role effects, where dorsal parts of mPFC (where our seed was located) were not engaged when responding to joint attention, but only during initiation (Redcay et al., 2012). However, ventral parts of mPFC have been found to respond during joint attention in one-person studies (Redcay et al., 2012; Schilbach et al., 2010), which we did not examine separately in our connectivity analysis due to our VOI selection approach (using a search mask around the group peak in mPFC).

The Bayesian model average included an inhibitory connection from mPFC on rTPJ in the receiver. MPFC signaling is therefore involved in the downregulation of rTPJ activity during joint attention. Interestingly, a similar inhibitory modulation of rTPJ through mPFC was also found for the sender, but only in the second block of the experiment. This is the only parameter showing differences when comparing the first and the last block. Speculatively, the modulation in the second block indicates learning within dyads, in the sense that after gathering experience with the task partner, a downregulation of rTPJ activity represents a more parsimonious network engagement.

### 4.5 Limitations and further notes

Notably, we focused on rTPJ function based on prior work, and included mPFC due to its central involvement in many social processes, including those engaged by our task. Any (h-) DCM result is conditioned on the model space considered. Hence, our result was the “best” fit for the model considered. Depending on the motivation of a particular study, other brain regions could be of interest. For example, one might be interested in modeling the processing of visual stimuli in more detail, and therefore include visual stream areas in their model; others might be interested in the role of reward in successful social interaction, which speaks to modeling of striatal responses; if affect is focused, the amygdala can be included in the network; and so on. Similarly, it remains unclear what role between-brain connectivity plays in settings that are not fully cooperative or even competitive, and future work probing more complex social functioning may result in different network architectures and “best” models. Related to this, DCM is a method for testing hypotheses. Analysis therefore begins by setting out a series of candidate network structures, which are then formalized as models. The method is therefore not suited for exploratory work, for which we would recommend other analyzes, such as a data-driven ICA and permutation for hyperscanning data (Bilek et al., 2015). Concluding, any choice on network structure (including seeds and their connections) must be justified by means of informed hypotheses about brain function and tested for relevance for the current data by means of model evidence or similar measures.

Our joint attention task probed a simple form of interpersonal exchange. Consequently, subjects performed the task very successfully and the behavioral data showed a clear ceiling effect. Future task designs may allow a meaningful relation of task performance and neural signatures. Additional, non-neuroimaging data may also prove useful in explaining interpersonal dynamics within the model, for example eye gaze behavior. Similarly, novel approaches to assess interactional behavior have proven useful sources to quantify social behavior and interactions (e.g., proxemic behavior patterns; Lahnakoski et al., 2020). However, such extensions of the data space result in an overwhelming complexity of the data, effects, and interactions, which suggests the employment of computational modeling to account for the rich multi-level data social interaction studies potentially provide. Such modeling has been proposed, among others, by Bolis and Schilbach (2017) for multi-subject data in the form of an active inference model, which could describe between-brain effects during social interaction, but also interpersonal coupling of behavior and other data. This would include a generative model of all levels of social exchange, for example, individual beliefs and expectations would be explicitly modeled, as well as learning, sensory experiences and observations, a representation of the interaction partner, but also a “model of the model” of the partner, and so on. Trial-wise predictions from their model, or any other computational model, could be gracefully integrated into h-DCM as regressors that drive or modulate two-person neural networks.

A number of methods have been used for the analysis of hyperscanning data, including functional connectivity (Koike et al., 2015), wavelet coherence (Goelman et al., 2019), independent component analysis (Bilek et al., 2015), and Granger causality (Schippers et al., 2010). These have been reviewed elsewhere (e.g., Czeszumski et al., 2020; Friston et al., 2013; Friston et al., 2014a; Misaki et al., 2021). Notable differences of h-DCM to other approaches can be summarized as follows: DCM is based on a generative model of fMRI data that examines brain networks on the level of neural activity, distinguishing neural effects from hemodynamics and the generation of the BOLD signal. While correlation provides a description of BOLD timeseries, DCM explains how these correlations were generated. Importantly, by scoring evidence for competing models, we explicitly test whether a connection between brains is necessary to explain the data or not, while controlling for shared perceptual input to both participants. Models are chosen based on model evidence, which is the trade-off between accuracy and complexity, rather than accuracy alone. This ensures that the simplest explanation for the data is selected that explains the most variance. H-DCM is applicable to all neuroimaging modalities, and may be complemented by further computational work, such as predictive coding models.

## 5 Conclusion

Using h-DCM we examined the effective connectivity between brain systems that are dynamically coupled during interaction. Our winning model included a causal impact of the sender's brain activity on the receiver's, which underlies previous reports of two-brain synchrony. In short, h-DCM may aid a mechanistic explanation of between-brain function based on generalized synchrony, which enables the discovery of between-brain connections in many forms of social contact.

## CRediT authorship contribution statement

**Edda Bilek:** Conceptualization, Formal analysis, Funding acquisition, Investigation, Methodology, Project administration, Writing – original draft, Writing – review & editing. **Peter Zeidman:** Formal analysis, Methodology, Writing – original draft, Writing – review & editing. **Peter Kirsch:** Conceptualization, Funding acquisition, Writing – review & editing. **Heike Tost:** Supervision, Writing – review & editing. **Andreas Meyer-Lindenberg:** Conceptualization, Funding acquisition, Supervision, Writing – review & editing. **Karl Friston:** Formal analysis, Methodology, Supervision, Writing – original draft, Writing – review & editing.

## Declaration of Competing Interest

A.M.-L. received the following incomes: Consultancy: Boehringer Ingelheim, Elsevier, Brainsway, Lundbeck Int. Neuroscience Foundation, Lundbeck A/S, The Wolfson Foundation, Bloomfield Holding Ltd, Shanghai Research Center for Brain Science, Thieme Verlag, Sage Therapeutics, v Behring Röntgen Stiftung, Fondation FondaMental, Janssen-Cilag GmbH, MedinCell, Brain Mind Institute, Agence Nationale de la Recherche, CISSN (Catania Internat. Summer School of Neuroscience), Daimler und Benz Stiftung, American Association for the Advancement of Science. Speaker fees: Italian Society of Biological Psychiatry, Merz-Stiftung, Forum Werkstatt Karlsruhe, Lundbeck SAS France, BAG Psychiatrie Oberbayern, Klinik für Psychiatrie und Psychotherapie Ingolstadt, med Update GmbH, Society of Biological Psychiatry, Siemens Healthineers. Written: A.M.-L. has received consultant fees from Boehringer Ingelheim, Elsevier, Brainsway, Lundbeck Int. Neuroscience Foundation, Lundbeck A/S, The Wolfson Foundation, Bloomfield Holding Ltd, Shanghai Research Center for Brain Science, Thieme Verlag, Sage Therapeutics, v Behring Röntgen Stiftung, Fondation FondaMental, Janssen-Cilag GmbH, MedinCell, Brain Mind Institute, Agence Nationale de la Recherche, CISSN (Catania Internat. Summer School of Neuroscience), Daimler und Benz Stiftung and American Association for the Advancement of Science and speaker fees from the Italian Society of Biological Psychiatry, Merz-Stiftung, Forum Werkstatt Karlsruhe, Lundbeck SAS France, BAG Psychiatrie Oberbayern, Klinik für Psychiatrie und Psychotherapie Ingolstadt, med Update GmbH, Society of Biological Psychiatry, Siemens Healthineers. All other authors declare no interst.
